# The Effects of the Contact Activation System on Hemorrhage

**DOI:** 10.3389/fmed.2017.00121

**Published:** 2017-07-31

**Authors:** Fabrício Simão, Edward P. Feener

**Affiliations:** ^1^Research Division, Vascular Cell Biology, Joslin Diabetes Center, Harvard Medical School, Boston, MA, United States

**Keywords:** kallikrein–kinin system, hemorrhage, hemostasis, factor XII, plasma kallikrein, contact activation system, coagulation

## Abstract

The contact activation system (CAS) exerts effects on coagulation *via* multiple mechanisms, which modulate both the intrinsic and extrinsic coagulation cascades as well as fibrinolysis and platelet activation. While the effects of the CAS on blood coagulation measured as activated partial thromboplastin time shortening are well documented, genetic mutations that result in deficiencies in the expression of either plasma prekallikrein (PPK) or factor XII (FXII) are not associated with spontaneous bleeding or increased bleeding risk during surgery. Deficiencies in these proteins are often undiagnosed for decades and detected later in life during routine coagulation assays without an apparent clinical phenotype. Increased interest in the CAS as a potentially safe target for antithrombotic therapies has emerged, in large part, from studies on animal models with provoked thrombosis, which have shown that deficiencies in PPK or FXII can reduce thrombus formation without increasing bleeding. Gene targeting and pharmacological studies in healthy animals have confirmed that PPK and FXII blockade does not cause coagulopathies. These findings support the conclusion that CAS is not required for hemostasis. However, while deficiencies in FXII and PPK do not significantly affect bleeding associated with peripheral wounds, recent reports have demonstrated that these proteins can promote hemorrhage in the retina and brain. Intravitreal injection of plasma kallikrein (PKal) induces retinal hemorrhage and intracerebral injection of PKal increases intracranial bleeding. PPK deficiency and PKal inhibition ameliorates hematoma formation following cerebrovascular injury in diabetic animals. Moreover, both PPK and FXII deficiency are protective against intracerebral hemorrhage caused by tissue plasminogen activator-mediated thrombolytic therapy in mice with thrombotic middle cerebral artery occlusion. Thus, while the CAS is not required for hemostasis, its inhibition may provide an opportunity to reduce hemorrhage in the retina and brain. Characterization of the mechanisms and potential clinical implications associated with the effects of the CAS on hemorrhage requires further consideration of the effects of PPK and FXII on hemorrhage beyond their putative effects on coagulation cascades. Here, we review the experimental and clinical evidence on the effects of the CAS on bleeding and hemostatic mechanisms.

## Introduction

**Figure 1:**
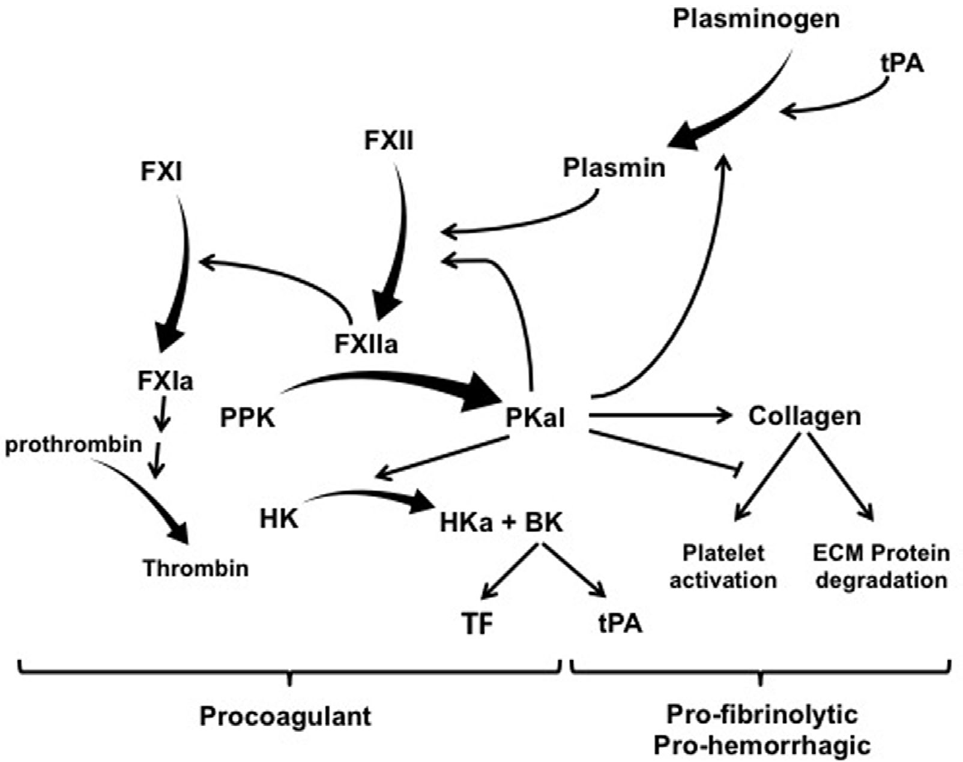
The contact activation system has multiple effects on coagulation, hemorrhage, and fibrinolysis. Exogenous tPA activates plasminogen and thereby mediated both plasmin-mediated fibrinolysis and activation of factor XII (FXII). FXIIa mediates its procoagulant effects by cleaving factor XI (FXI) into FXIa, which leads to the generation of thrombin. FXIIa also cleaves PPK into PKal, which exerts a combination of effect on thrombosis, fibrinolysis, and hemorrhage. PKal’s effects on hemorrhage have been attributed to its direct and indirect effects on collagen cleavage. In addition, PKal can also interferes with collagen-induced platelet activation, which can impair hemostasis. Activation of the kallikrein–kinin system generates bradykinin, which stimulates expression of both TF and tPA. Abbreviations: HK, high molecular weight kininogen; PPK, plasma prekallikrein; PKal, plasma kallikrein; BK, bradykinin; ECM, extracellular matrix; tPA, tissue plasminogen activator; TF, tissue factor.

The contact activation system (CAS) represents a group of plasma proteins, including factor XII (FXII), plasma prekallikrein (PPK), and high molecular weight kininogen (HK) that promotes inflammation and coagulation upon contact of blood with an activating surface or protease (1, 2). CAS activation is initiated by interactions of FXII with a negatively charged surface or a protease that induces a conformational change in FXII leading to its proteolytic cleavage and the generation of the serine protease FXIIa (3, 4). The two primary substrates for FXIIa are PPK and factor XI (FXI). FXIIa-mediated cleavage of PPK results in its zymogen activation to the serine protease plasma kallikrein (PKal), which converts FXII to FXIIa and thereby provides positive feedback amplification of the CAS. The effects of the CAS on inflammation, vascular permeability, and edema are primarily attributed to the Kallikrein–Kinin System. This system involves PKal-mediated cleavage of HK to generate the nonapeptide hormone bradykinin (BK), which activates B2 receptors (B2R) that are expressed on a variety of vascular, neuronal, and immune cell types. Binding of BK to B2R activates proinflammatory signaling pathways that dilate vessels, induce chemotaxis of neutrophils, and increase vascular permeability (5, 6). C1-inhibitor (C1-INH) is the primary physiological inhibitor of both FXIIa and PKal, and C1-INH deficiency facilities CAS activation and BK-mediated angioedema (7). Both PKal and the B2R are clinically significant mediators of hereditary angioedema (HAE) (8, 9). In addition to its effects on inflammation and vascular permeability, BK has been shown to induce expression of both tissue factor (TF) and tissue plasminogen activator (tPA), which activates the extrinsic coagulation pathway and fibrinolysis, respectively (10–13) (Figure 1).

The acute effects of the CAS on coagulation have been primarily attributed to FXIIa-mediated activation of FXI and thereby the intrinsic coagulation pathway leading to fibrin generation. Although the biochemical roles of PKal and FXIIa as upstream triggers for the intrinsic coagulation system were recognized over 50 years ago (14), the clinical significance of these factors on thrombosis and hemorrhage is not fully understood. It is well documented that CAS blockade results in prolonged coagulation times in the activated partial thromboplastin time (aPTT) assay (15). The absence of thrombotic and hemostatic abnormalities in individuals with genetic PPK or FXII deficiency has suggested that the CAS plays a minimal role in physiological coagulation. However, information from rare case studies of individuals with deficiencies in individual components of the CAS has provided limited insight into the potential clinical significance of PKal and FXIIa in coagulation in specific diseases. This is further complicated by the interpretation of acquired and mild CAS component deficiencies, which may reflect the activation of the system rather than its suppression. Key observation from studies of humans with genetic FXII and PPK deficiencies is the absence of spontaneous bleeding disorders and increased bleeding risk during surgery, which are observed in individuals with genetic mutations in downstream in the intrinsic pathway, such as FIX (hemophilia B) and FXI (hemophilia C). The main evidence suggesting that CAS inhibition can reduce pathological thrombosis without increasing bleeding has emerged from studies using PPK and FXII-deficient animals with acute and artificially provoked coagulation (16–20). These clinical and experimental findings have generated increased interest in the potential therapeutic opportunities of targeting the CAS in thrombosis. Indeed the observations of the blockade of the CAS results in reduced thrombosis without a concomitant increase in bleeding in preclinical studies have suggested that therapies that inhibit the CAS may offer novel strategies for safe and effective antithrombotics. Moreover, recent studies suggest that blockade of the CAS can improve hemostasis in the retina and brain. In this review, we discuss emerging understanding of the role of CAS on bleeding and hemostasis and the clinical implications of this research.

## Hemostasis

Hemostasis is the process that prevents blood loss following vascular injury. The mechanisms of hemostasis involve both serial and parallel events, mediated by the interactions between circulating factors in blood with components of activated or injured vascular tissue. Given the critical nature of hemostasis, the system has evolved both redundant and reinforcing mechanisms that facilitate both rapid and stable cessation of blood loss. The sequence of events and the relative contributions and requirements of individual coagulation components may vary according to the affected tissue and type of injury, and possibly also the animal species. While thrombosis and hemostasis share a host of coagulation factors, the etiology of these processes has important distinctions, which may create therapeutic opportunities to selectively target thrombosis without affecting hemostasis.

Although animal models do not fully recapitulate the complexity of human physiology and disease, studies on animals with targeted deficiencies in coagulation factors have provided insight into contributions of individual components of coagulation in hemostatic mechanisms. Multiple approaches are used to quantify hemostatic responses in rodents, including tail tip bleeding, cuticle bleeding, and tail vein transection assays. Deficiencies in FXII and PPK in healthy animals exert little or no effect on tail bleeding time despite aPTT prolongation (Table 1), in contrast to prothrombin deficiency, which markedly increases blood loss in tail wound models (21).

**Table 1:** Effects of contact activation system inhibition and deficiencies in experimental models.

Condition	aPTT	Tail bleeding	Spontaneous bleeding	Species	Reference
FXII^−/−^	Prolonged	No changes	Not reported	Mice	Renne et al. (16)
FXII^−/−^	Prolonged	No changes	Not reported	Mice	Iwaki et al. (22)
Anti-FXII (9A2 and 15H8)	Prolonged	No changes	No	Primate	Matafonov et al. (23)
FXII ASO	Not reported	No changes	No	Mice	Revenko et al. (24)
FXII^−/−^ or Infestin-4	Prolonged	No changes	No	Mice	Nickel et al. (25)
Infestin-4	Prolonged	No changes	No	Mice	Hagedorn et al. (26)
FXII^−/−^ or FXII sRNAi	Prolonged	No changes	No	Rat	Cai et al. (27)
PPK ASO	Not reported	No changes	No	Mice	Revenko et al. (24)
Klkb1^−/−^	Prolonged	No changes	Not reported	Mice	Liu et al. (17)
PPK ASO	Prolonged	No changes	No	Mice	Bird et al. (18)
Kng1^−/−^	Prolonged	No changes	Not reported	Mice	Merkulov et al. (28)
Bdkrb2^−/−^	Prolonged	Prolonged	Not reported	Mice	Shariat-Madar et al. (29)

*ASO, antisense oligonucleotide; aPTT, activated partial thromboplastin time; infestin-4 is an FXIIa inhibitor; 9A2 and 15H8 are anti-FXII inhibitory antibodies; FXII, factor XII; PPK, plasma prekallikrein*.

Deficiencies in downstream factors in the intrinsic cascade (such as FIX and FXI) result in bleeding disorders, demonstrating their role in physiological hemostasis. Paradoxically, deficiencies in the CAS factors FXII, PPK, or HK, which trigger coagulation *in vitro* and contribute to thrombosis in animal models, are not associated with a bleeding diathesis. However, while surgical interventions provide a general assessment of hemostasis, it does not fully model the potential contributions of the CAS in all hemorrhagic conditions. Indeed, recent studies have suggested that the CAS may play an important role in hemorrhage in the retina and brain in certain diseases (17, 20, 30).

### Effects of Kallikrein–Kinin System on Platelet Activation

The initial steps in hemostasis involve vasocontraction and the local aggregation of platelets to rapidly restrict bleeding at the site of endothelium disruption. Vascular spasm and vasoconstriction, which reduces local blood flow, is triggered by factors released from vascular smooth muscle, endothelial cells, and platelets, and reflexes initiated by the local sympathetic nervous system. Platelets adhere *via* glycoprotein VI (GPVI) receptors to exposed basement membrane collagen at the site disrupted endothelium to initiate platelet aggregation. This process leads to tethering and activation of platelets, additional platelet receptor-collagen interactions, and platelet plug formation. GPVI play a critical role in normal hemostasis, and its deficiency results in abnormal platelet-responses to collagen and mild bleeding tendency (31).

A report by Liu et al. (17) has shown that PKal inhibits collagen-induced platelet aggregation. PKal also interferes with platelet aggregation induced by collagen-related peptide, a GPVI agonist, but not by convulin; a snake venom toxin that is a collagen-independent GPVI agonist. Moreover, PKal does not alter either thrombin’s or ADP’s effects on platelet aggregation. These findings suggest that PKal’s effects on platelet activation were specific to GPVI interactions with collagen. Interestingly, the inhibitory effects of PKal on collagen-induced platelet activation were not mimicked with PPK, suggesting that the activate form of this protein is required for its inhibitory effect. These findings suggest that PKal binding to collagen in the basement membrane at the site of endothelium disruption interferes with GPVI-mediated platelet activation during initial stages of hemostasis. PKal binding to collagen and its effects on platelet aggregation are increased at elevated glucose concentrations, suggesting that hyperglycemia enhances PKal inhibition of collagen-induced platelet aggregation and thereby may contribute to hematoma expansion in diabetes. Although the mechanism by which glucose increases the binding of collagen to PKal has not been identified, previous reports have suggested that an exposure of collagen to hyperglycemia can alter collagen conformation and facilitate protein binding (32). Interestingly, hyperosmolar concentrations of mannitol, a treatment commonly used to reduce brain edema following stroke, increased hematoma expansion in non-diabetic rats and enhanced PKal’s inhibitory effects on collagen-induced platelet aggregation *in vitro*. These results suggest that plasma osmolality may alter PKal’s effects on coagulation. The mechanism of PKal interference of collagen-platelet GPVI binding appears analogous to the effects of anopheline antiplatelet protein in the saliva of the malaria vector mosquito, which also interferes with collagen-induced platelet activation and hemostasis (33). Further studies are needed to map the domain on PKal that binds to collagen and the factors that modulate this binding. In addition to altering platelet interactions, PKal binding to collagen may retain this enzyme at the site of vascular injury to facilitate its local inflammatory effects. In contrast to the inhibitory effects of PKal on hemostasis, FXII activation by collagen and laminin in the subendothelial matrix can lead to thrombin generation, which may promote secondary mechanisms of platelet activation at the site endothelium disruption (34, 35). Although these findings suggest that the CAS can exert both positive and negative effects on platelet activation during hemostasis, gene targeting and pharmacological studies indicated that blockade of CAS reduces cerebral hemorrhage, which is a primary area of concern regarding bleeding in patients on antithrombotic therapy.

### Effects of Kallikrein–Kinin System on Fibrin Formation

Stabilization of the nascent thrombus involves additional coagulation factors that generate and crosslink fibrin, which stabilizes nascent platelet plugs. FXIIa-mediated activation of FXI results in the activation of the intrinsic coagulation cascade, leading to thrombin activation and fibrinogen cleavage and the stabilization and expansion of clots. Although FXIIa can serve as a trigger to activate the intrinsic pathway from the CAS, positive feedback activation of FXI by thrombin can also activate this pathway following extrinsic activation (36–39). Thus, while FXII appears to contribute to arterial thrombus formation and growth, at least in animal models, FXII does not appear to be required for clot formation during hemostasis. Activated platelets actively contribute to this secondary hemostatic process by generating surface-bound polyphosphate nanoparticles (40), which activates FXII and thereby promotes local fibrin generation by the clot. Although FXII, PPK, or HK deficiency is not associated with bleeding, FXI deficiency in patients is associated with mild bleeding, suggesting that the role of FXI in hemostasis is independent of the CAS. The upstream activation of FXI by thrombin may explain why FXII is not required for hemostasis in contrast to FXI, where its deficiency is associated with hemophilia C.

Clinical trials and epidemiologic data indicate that FXI contributes to thromboembolic diseases. Studies with animal models also suggest that the activation of FXI by FXIIa promotes pathological thrombus formation (41). Furthermore, “exposed” collagen also initiates the contact phase of coagulation by binding to FXII and enhances coagulation *in vitro* (35). The conversion into active FXIIa is dependent on repetitive negative charge exposed by collagen fibrils (42). van der Meijden and colleagues (43) showed that collagen potentiates thrombus formation *via* binding to FXII, leading to its activation and subsequent FXI activation. This report suggests a dual role for collagen in thrombin generation by stimulating platelet activation *via* GPVI and by direct effects on FXII. In addition, FXIa-mediated thrombin activation may contribute to clot stability thrombin-activatable fibrinolysis inhibitor-mediated removal of lysine residues on fibrin, which increases its resistance to fibrinolysis (44). Moreover, FXIa proteolytically degrades and neutralizes TF pathway inhibitor, which can promote thrombin generation *via* the extrinsic pathway (45). Recently, Stavrous and colleagues (13) demonstrated that PPK deficiency in mice increase prostacyclin through Sirt1 and KLF4, which reduced vascular TF expression and thrombosis. Thus, while CAS’s effects on both the intrinsic and extrinsic pathways contribute to clot stabilization, mechanistic redundancy contributing to fibrin generation minimizes the requirements of the CAS in this process.

## Effects of PPK, FXII, and C1-INH Deficiency on Coagulation in Humans

Most reports have indicated that individuals with severe deficiencies in either PPK or FXII do not display abnormalities in either thrombosis or bleeding (Table 2), and often go undiagnosed for decades without apparent clinical phenotype. The absence of a bleeding diathesis in FXII and PPK deficiencies appears to contradict the key role of these proteins in coagulation *via* the intrinsic pathway *in vitro*, and this has left the physiological significance of the CAS in coagulation and thrombosis unclear. However, CAS could be a trigger for thrombosis associated with extracorporeal circuits (46) and cardiopulmonary bypass (47) by exposing blood to artificial surfaces, non-physiological shear stress, and osmotic forces. Administration of an antibody that neutralizes FXIIa (3F7) has been shown to have a similar effect as heparin in preventing thrombosis during extracorporeal circuits (48).

**Table 2:** Case reports of deficiencies in contact activation system proteins.

Deficiency	aPTT	Thrombosis and clinical presentation	Spontaneous bleeding	Reference
FXII	Prolonged	Bilateral femoral vein thrombosis	No	Cei et al. (49)
FXII (mild)	Slightly prolonged	Venous thrombosis	No	Lessiani et al. (50)
FXII	Prolonged	Coronary artery bypass grafting	No	Conaglen et al. (51)
FXII	Prolonged	Arterial and venous thrombosis	No	Hellstern et al. (52)
FXII	Prolonged	Coronary artery bypass grafting	No	Moorman et al. (53)
FXII (mild)	Prolonged	Occlusive thrombus in the circumflex and anterior descending arteries	No	Penny et al. (54)
FXII	Prolonged	Coronary artery bypass grafting	No	Rygal and Kuc (55)
FXII	Prolonged	Coronary artery disease	No	Cronbaugh et al. (56)
FXII	Prolonged	Coronary artery bypass grafting	No	Salmenper et al. (57)
FXII	Prolonged	No thrombosis	No	van Veen et al. (58)
FXII	Prolonged	Coronary artery disease	No	Wood (59)
FXII (mild)	Prolonged	Bilateral lower limb deep vein thrombosis	No	Vergnes et al. (60)
FXII	Prolonged	Retinal venous thrombosis	No	Borrego-Sanz et al. (61)
FXII (mild)	Prolonged	Deep vein thrombosis, after abdominal surgery	No	Cornudella et al. (62)
FXII	Prolonged	Cardiopulmonary bypass	No	Gerhardt et al. (63)
PPK	Prolonged	No thrombosis	No	van Veen et al. (58)
PPK	Prolonged	Coronary artery disease	No	Oram et al. (64)
PPK	Prolonged	No thrombosis	No	Cankovic et al. (65)
PPK	Prolonged	No thrombosis	No	Asmis et al. (66)
PPK	Prolonged	No thrombosis	No	Maak et al. (67)
PPK	Prolonged	No thrombosis	No	DeLa Cadena (68)
PPK	Prolonged	Ischemic stroke	No	Francois et al. (69)
PPK	Prolonged	No thrombosis	Idiopathic thrombocytopenic purpura	Nakao et al. (70)
PPK	Prolonged	No thrombosis	No	Lombardi et al. (71)
PPK	Prolonged	No thrombosis	No	Wynne Jones et al. (72)
PPK	Prolonged	No thrombosis	No	Poon et al. (73)
HK	Prolonged	No thrombosis	No	Cankovic et al. (65)
HK	Prolonged	Cardiopulmonary bypass	No	Davidson et al. (74)
HK	Prolonged	No thrombosis	No	Lefrere et al. (75)
HK	Prolonged	No thrombosis	No	Stormorken et al. (76)
HK	Prolonged	Vertebral-basilar artery thrombosis following trauma	No	Krijanovski et al. (77)

*aPTT, activated partial thromboplastin time; FXII, factor XII; PPK, plasma prekallikrein; HK, high molecular weight kininogen; NA, not available*.

*Mild, 50 to 20% of control*.

Activation of FXII by kaolin (negatively charged aluminum silicate particulate) provides the basis for the aPTT, a clinical clotting assay, which is widely used to assess the integrity of the intrinsic pathway and to monitor anticoagulation with heparin. Deficiencies in FXII, PPK, and HK results in markedly prolonged aPTT and no increases in spontaneous bleeding or impairment in hemostasis (Table 2). Normalization of a severely increased aPTT (>120 s) after prolonged preincubation with aPTT reagent occurred in plasma deficient in PPK but not in plasma deficient in FXII, HK, FXI, FIX, FVIII, and Passovoy trait plasma (a deficiency characterized by abnormal coagulation affecting the intrinsic coagulation system) or plasma containing lupus anticoagulant. Autoactivation of FXII in PPK-deficient plasma in the presence of kaolin paralleled the normalization of aPTT. The addition of OT-2, a monoclonal antibody neutralizing FXII, prevents the normalization of aPTT (66). These results suggest that autoactivation of FXII is responsible for normalization of a severely prolonged aPTT upon increased preincubation time in PPK-deficient plasma.

In humans, PPK deficiency is not associated with hemostatic disorders, indicating PKal is not required for hemostasis. Although some case reports have associated FXII deficiency with prothrombotic events, a clinical study failed to demonstrate a significant correlation between increased risk of thrombosis and FXII deficiency (78). Girolami and colleagues (79) re-evaluated case reports on FXII deficiency and thrombosis. They showed that, in most cases, FXII deficiency was associated with other congenital or acquired prothrombotic risk factors. While these studies indicate that the CAS is not essential for either thrombosis or hemostasis, individuals with genetic deficiencies in the CAS are rare and it is not possible to ascertain from the anecdotal clinical information whether PKal and FXIIa could have significant roles in thrombosis or hemostasis associated with specific clinical indications.

C1-inhibitor is the primary endogenous inhibitor of both PKal and FXIIa. HAE is a rare genetic disease caused by deficiencies in C1-INH concentration (Type I) or C1-INH activity (Type II) (80). HAE has a prevalence of approximately 1:50,000 and is characterized by episodes of vasogenic edema, which vary in severity and affected tissue, and can become life threatening when affecting the larynx. The primary mechanism for this disease is the uncontrolled activity of PKal, which results in increased cleavage of HK, and increased production of BK. While the molecular and physiological triggers for the onset of attacks are not fully understood, increased levels of FXIIa is observed in this disease (81) which suggest that attacks are mediated by the CAS. Since HAE is not associated with coagulopathies, this disorder provides an opportunity to evaluate the effects of the CAS on downstream coagulation pathways, independent of confounding input associated with comorbidities of thrombosis and hemostasis. Indeed, plasma obtained from HAE subjects during edematous attacks display shortened aPTT, compared to plasma collected from these subjects during remission (82, 83). In addition, FXIa activity, prothrombin fragment F1 + 2, thrombin-anti-thrombin complex, and FVIIa are elevated in subjects with HAE during an attack (81, 83, 84). The absence of apparent clinical coagulopathies in HAE during attacks could be due to compensatory mechanisms that occur during attacks and/or requirements of additional factors not activated by the CAS to initiate or promote thrombosis formation.

## Effects of PPK and FXII Deficiency on Coagulation in Rodents

Consistent with PPK and FXII deficiencies in humans, PPK, and FXII-deficient mice display prolonged aPTT (Table 1). Pharmacological inhibition of FXIIa and deficiency in either FXII or HK genes have been shown to protect mice from experimentally induced thrombosis (16, 24, 26, 28), indicating the potential role of the CAS in thrombotic disease. Cheng and colleagues (41) demonstrated that a neutralizing antibody to FXI (14E11) had a comparable effect to FXI deficiency in a FeCl_3_ model of thrombosis. Antibody 14E11 binds FXI and interferes with FXI activation by FXIIa, suggesting that thrombus formation in this model requires FXI activation by FXIIa. These data have generated interest in developing strategies to therapeutically inhibit FXIIa and contact activation to treat or prevent thromboembolic disorders. Interestingly, thrombosis protection in mice was greater with FXII deficiency than with FXI deficiency, suggesting that FXII may exert effects on coagulation that do not require FXI (41).

Factor XII-deficient mice showed defective thrombus formation (16) and protective effect against experimental ischemic stroke (85) and pulmonary embolism (86). Intravital microscopy showed that the initial adhesion of platelets at the site of injury is not affected by FXII deficiency, although the formation and stabilization of three-dimensional thrombi is impaired (16). Similar defective thrombus formation was observed in FXI^−/−^ mice (87, 88). Furthermore, FXII deficiency or pharmacological inhibitor of FXIIa showed decreased infarct volume without the presence of intracerebral hemorrhage (ICH) (85). Reconstitution of FXII-deficient mice with human FXII protein restored susceptibility for ischemic stroke. A recent report by Nickel and colleagues (25) has shown that prostasomes released from prostate cancer activated FXII and that both FXI- and FXII-deficient mice are protected against cancer-associated thrombosis (25). However, the observation that PPK-deficient mice were not protected in the prostasome-induced thrombosis model is surprising and appears different than other CAS-associated thrombosis models, which require PPK. Taken together, these findings suggest that coagulation mediated by intrinsic pathway through FXII is critical for pathological thrombus formation in certain conditions without being relevant for hemostasis.

In addition, the CAS mediates effects on thrombosis *via* the kallikrein–kinin system. Genetic ablation of Kng1 in mice showed a delayed time to carotid artery occlusion in a laser injury model (28). BKB2R^−/−^ mice have prolonged bleeding time and delayed carotid artery occlusion times in the rose Bengal thrombosis model and these effects were attributed to increased expression of angiotensin receptor 2 and elevated nitric oxide and prostacyclin levels (29). These findings suggest that the BK system contributes to the effects of the CAS on coagulation.

## Potential Role of the Kallikrein–Kinin System in Hemorrhage

Components of the CAS are normally restricted from contact with the vascular basement membrane by the endothelium. Vascular hyperpermeability enables components of the CAS to leak into the subendothelial space and gain contact with the basement membrane. The potential functions of the CAS in the subendothelial space have received relatively little attention. PKal binds to collagen (89), which may contribute to the retention of its catalytic activity in the extracellular matrix (ECM) at the site of injury and thereby enable local amplification of its inflammatory and edematous actions. Moreover, PKal in this subendothelial space may have additional substrates and functions, which are not apparent in the plasma.

### Diabetic Retinopathy

In diabetic retinopathy, retinal vascular hyperpermeability can lead to diabetic macular edema (DME); the leading cause of vision loss in working-age adults in most developed countries (90). Proteomic analysis of vitreous fluid from patients with DME has revealed increased concentrations of PPK, HK, and FXII in this fluid compared with people without DME (91–93). These increases have been attributed to the increased diffusion of these CAS components from the blood into the neuroretinal interstitial fluid and vitreous humor. Recent reports have shown that intravitreal injection of PKal alters both retinal vascular function and ultrastructure. A single injection of PKal in the vitreous of rats induced retinal vascular hyperpermeability and retinal layer thickening (94), whereas two injections of purified PKal into the vitreous caused retinal bleeding that appeared similar to retinal microhemorrhages that occur in diabetic retinopathy (30). While injections of BK mimicked PKal’s effect on retinal permeability and thickening at 24 h repeated injections of BK did not cause retinal bleeding at 48 h. These findings suggest that PKal in the subendothelial space may contribute to retinal hemorrhages, which are a hallmark of advanced stages of diabetic retinopathy. Proteomics has been used to investigate the effects of PKal that may contribute to this disruption of the blood–retinal barrier. Analyses of conditioned media from both cultured pericytes and astrocytes incubated with PKal have revealed increases in proteolytic fragments of ECM proteins, including multiple collagen isoforms 1–6, laminin β1 and γ1, nidogen 1 and 2, and fibronectin, compared with conditioned media from cells that were not exposed to PKal (30, 95). In a purified system, PKal was shown to cleave COL4A, suggesting that PKal can proteolytically cleave collagen (30). Although the causes of retinal bleeding in diabetic retinopathy are not yet known, these studies suggest a mechanism by which vascular permeability, *via* the extravasation of PKal, may contribute to retinal hemorrhage, in part, mediated by the proteolytic degradation of the vascular basement membrane.

### Cerebral Hematoma Formation

Diabetes and hyperglycemia are associated with increased ICH and worse clinical outcomes following a cerebrovascular accident (96–98). Diabetes and acute hyperglycemia in non-diabetic rodents increase hematoma formation in an experimental model of ICH (17). In this report by Liu et al., intracerebral injection of autologous blood induced hematoma expansion, which was ameliorated by PPK deficiency and PKal inhibition. Moreover, intracerebral injection of purified PKal mimicked the effects of autologous blood injection on hematoma expansion in diabetic animals. PKal has been reported to mediate plasminogen activation (99, 100), which could potentially increase fibrinolysis in the ICH model. However, intracerebral injection of neither tPA nor plasmin mimicked the effects of PKal on hematoma expansion in diabetic animals (17). Moreover, covalently deactivated PKal also induced hemorrhage in this model, suggesting that PKal’s effects in this model were not mediated by its catalytic activity. The effects of hyperglycemia and PKal on ICH are rapid, within 30 min, suggesting an effect on an early step in hemostasis. Since *in vitro* studies have shown that PKal binding to collagen interferes with its effects on GPVI-mediated platelet activation, these findings suggest that PKal may decrease platelet plug formation, which plays an early step in hemostasis.

### Thrombolysis

Tissue plasminogen activator is the only approved treatment for thrombotic stroke (101). While its prompt administration of tPA following stroke onset has been shown to improve clinical outcomes, its use is limited due to an increased risk of intracranial hemorrhage when tPA is used after the recommended 3 h therapeutic window, which can negate the potential benefits of vascular recanalization. Although the mechanisms that mediate the increase in hemorrhage induced by tPA are not fully understood, we have shown that both PPK and FXII deficiency markedly reduce tPA-induced hemorrhagic conversion in mice with a thrombotic middle cerebral artery occlusion (20). Studies using purified proteins, as well as plasma, revealed that plasmin activates FXII and cleaves it into a fragment with a molecular weight slightly higher than the expected size of FXIIa light chain generated with PKal (20, 102–105). Plasmin cleaves FXII at Arg353 leads to zymogen activation and the generation of FXIIa (104). Plasmin’s effects on FXIIa activity is markedly increased in the presence of dextran sulfate (104) and inhibited by e-aminocaproic acid and a FXIIa inhibitory mAb (105). These reports have suggested that plasmin-mediated cleavage of FXII increases FXIIa-like activity. The consequences of plasmin-mediated cleavage of FXII at Lys346 on FXIIa-like catalytic activity are not yet available. Plasmin-mediated cleavage of FXII could facilitate its autoactivation and/or cleavage by PKal in plasma, which would thereby activate the kallikrein–kinin system.

These findings have suggested that the CAS may either worsen vascular damage or impair hemostasis in the cerebral vasculature following tPA administration. Studies using human plasma have shown that tPA’s effects on the CAS are mediated by plasmin (20), which has been identified as a physiological activator of the CAS (104, 105). Although the clinical significance of this pathway in hemorrhagic conversion in ischemic stroke is not yet available, two lines of evidence support the conclusion that tPA therapy activates the CAS in stroke patients. First, a recent report by Marcos-Contreras and colleagues has shown that intravenous infusion of tPA in patients result in plasma HK cleavage (106), suggesting that the tPA activates the circulating CAS. Second, tPA therapy can cause orolingual angioedema that has been attributed to increased BK action (107, 108, 109), suggesting that tPA increases CAS activity. In addition to reducing hemorrhage, PPK, and FXII deficiency also reduced infarct volume and cerebral edema in mice with stroke treated with tPA. These findings are consistent with previous reports showing that blockade of the CAS has neuroprotective effects in mice with filament-mediated middle cerebral artery occlusion (85, 110). Although it is tempting to speculate that inhibition of either PKal or FXIIa during the administration of tPA may ameliorate hemorrhagic transformation and provide neuroprotection during thrombolytic therapy, additional information on the role of the CAS on stroke outcomes in animal models is needed.

## Therapeutic Implications and Conclusion

Currently available anticoagulants used for prevention or treatment of thromboembolic events [heparins, vitamin K antagonists (for example, warfarin), and inhibitors of thrombin or factor Xa] all target enzymes of the coagulation cascade that are essential for the formation of fibrin, a protein necessary for controlling injury-related blood loss. As a result, currently used anticoagulants increase the risk of bleeding and are associated with an increase risk in potentially life-threatening hemorrhage (111). Bleeding is the primary complication of anticoagulation therapy and a significant risk of all currently used anticoagulants, even when maintained within their therapeutic ranges (112). The CAS exerts effects on coagulation at multiple levels. CAS activation of PPK has been implicated in promoting spontaneous microvascular bleeding and the impairment of collagen-induced platelet activation. In addition, both PPK and FXII, *via* activation of the intrinsic coagulation cascade promotes clot stabilization and growth. Feedback activation of FXI by thrombin contributes to clot stabilization and may explain the lack of dependence of hemostasis on FXII and PPK. PKal has been implicated in promoting spontaneous microvascular bleeding and the impairment of collagen-induced platelet activation. Pharmacological blockade of PKal has been shown to provide beneficial effects on cerebral hemostasis in animal models. Although inhibition of the CAS reduces provoked arterial and venous thrombosis in animal models, the clinical significance of the CAS in thrombosis is not yet available. The clinical indications for targeting the CAS for thrombosis will require the identification of thrombotic processes that are dependent on the CAS, which potentially include interactions with artificial surfaces and disease processes that generate factors that activate FXII. In addition, inhibition of the CAS may provide an opportunity to reduce cerebral hemorrhage, which is one of the primary concerns of increased bleeding risk associated with current antithrombotics.

## Author Contributions

FS and EF performed literature searches and wrote the manuscript.

## Conflict of Interest Statement

EPF is an employee of KalVista Pharmaceuticals Inc. (Cambridge, MA, USA). FS declares no competing financial interests.
